# Correction: Sato, K., et al. Identification of a Novel Oligosaccharide in Maple Syrup as a Potential Alternative Saccharide for Diabetes Mellitus Patients. *Int. J. Mol. Sci.* 2019, *20*, 5041

**DOI:** 10.3390/ijms21145159

**Published:** 2020-07-21

**Authors:** Kanta Sato, Noriaki Nagai, Tetsushi Yamamoto, Kuniko Mitamura, Atsushi Taga

**Affiliations:** Faculty of Pharmacy, Kindai University, 3-4-1 Kowakae, Higashi-Osaka, Osaka 577-8502, Japan; 2045110007u@kindai.ac.jp (K.S.); nagai_n@phar.kindai.ac.jp (N.N.); yamatetsu@phar.kindai.ac.jp (T.Y.); mitamura@phar.kindai.ac.jp (K.M.)

The authors wish to make the following corrections to this paper [1]:

We have found some errors in the NMR spectra data and structure of the compound; therefore, we wish to make corrections to Table 1, Figure 3, and the title, as well as to the following sections: Abstract, Introduction, Results, Discussion, and Material and Methods.

## 1. Change in Table 1

The authors wish to make the following correction to this paper [1]. Due to mislabeling, replace:

**Table 1:** ^1^H (800 Mz) and ^13^C (200 Mz) nuclear magnetic resonance spectral data for maplebiose1 in D_2_O.

Chemical Shift (ppm)			
Position	H (Proton)	C (Carbon13)	Intensity
1	4.4832	98.5740	57.4308
2	3.0938	76.6692	51.0309
3	3.3153	78.2276	64.6557
4	3.2818	72.2547	63.2886
5	3.3905	77.5813	39.4174
6	3.5403	62.7243	180.0717
7	3.5912	62.7459	58.2694
8	4.0197	79.5228	101.6335
9	3.9639	77.2054	62.9815
10	3.7169	83.8104	46.7190
11	3.8738	63.3779	34.1492

Chemical shifts are shown in ppm and coupling constants are shown in Hz in parentheses.

with

**Table 1:** ^1^H (800 MHz) and ^13^C (200 MHz) nuclear magnetic resonance spectral data for maplebiose1 in D_2_O.

Chemical Shift											
Residue	Position	δ_C_	δ_H_	*J*_H,H_	Type	Residue	Position	δ_C_	δ_H_	*J*_H,H_	Type
Glc α	1	94.72	5.07	3.8	d	Glc β	1	98.57	4.49	8.0	d
	2	74.05	3.38	3.8, 9.8	dd		2	76.67	3.09	8.0, 9.3	dd
	3	75.25	3.54	-	m		3	78.23	3.33	9.3, 9.3	dd
	4	72.32	3.29	9.0, 9.0	dd		4	72.25	3.29	9.0, 9.0	dd
	5	73.27	3.77	1.9, 9.2, 9.7	ddd		5	77.58	3.40	2.1, 8.7, 9.7	ddd
	6	63.38	3.82	2.1, 10.9	dd		6	63.38	3.87	2.0, 11.0	dd
Fru β	1’	62.75	3.60	-	m	Fru β	1’	62.72	3.54	-	m
	2’	106.31	-	-	-		2’	106.34	-	-	-
	3’	79.42	4.03	8.5	d		3’	79.52	4.02	8.5	d
	4’	77.06	3.97	8.1, 8.2	dd		4’	77.21	3.96	8.0, 8.3	dd
	5’	83.75	3.72	7.4	t		5’	83.81	3.72	7.4	t
	6’	64.98	3.54–3.66	-	m		6’	65.10	3.54–3.66	-	m

Chemical shifts (**δ_C_** and **δ_H_**) are shown in ppm and coupling constants (***J*_H,H_**) are shown in Hz. All δ_C_ values of maplebiose1 are close to those of blastose, although uniformly shifted by 0.2 ppm.

## 2. Change in Figure 3

The authors wish to make the following correction to this paper [1]. Due to mislabeling, replace:

**Figure 3:**
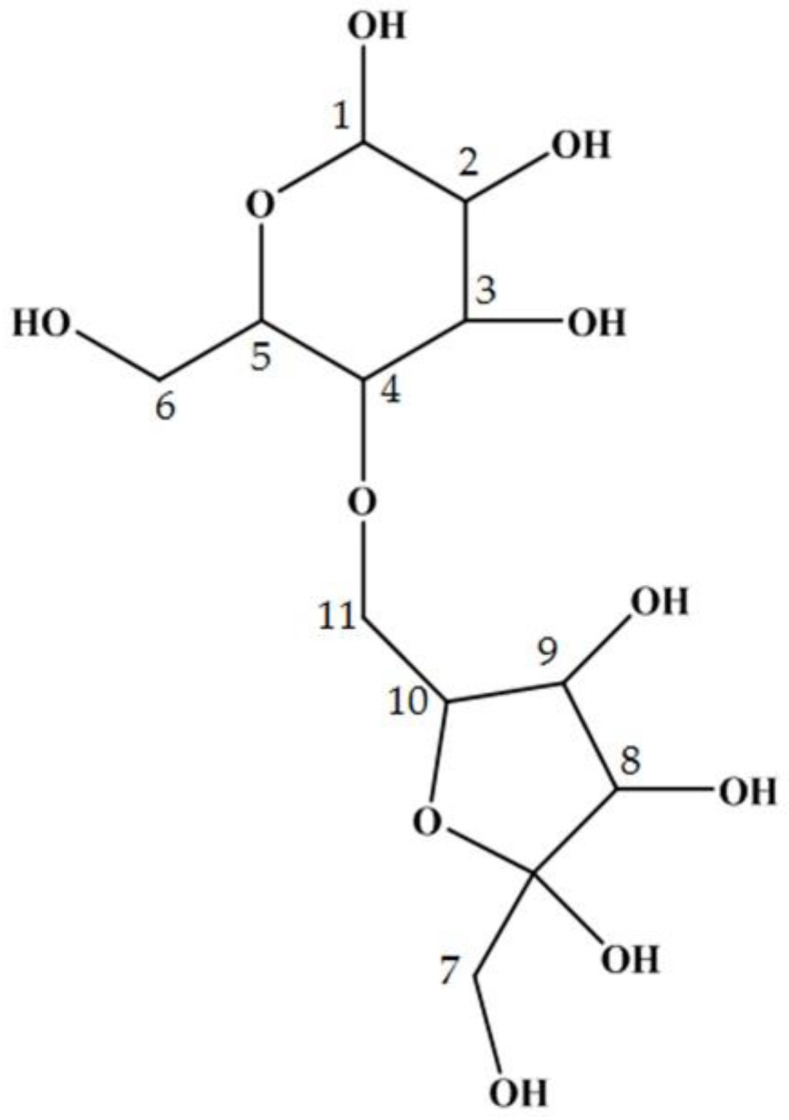
The structure of maplebiose1, obtained from maple sap. The proton and carbon signals for each number are listed in Table 1.

with

**Figure 3:**
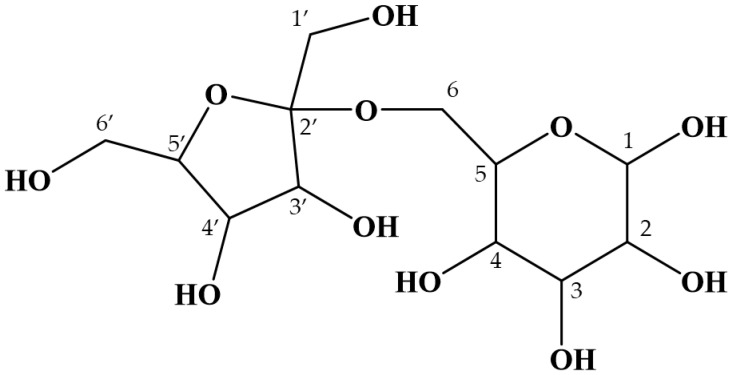
The structure of maplebiose1, obtained from maple sap. Maplebiose1 was identified as blastose. The proton and carbon signals for each number are listed in Table 1.

These corrections have induced a few major changes in the text of the title, as well as in the Abstract, Introduction, Results, Discussion, and Material and Methods sections.

## 3. Change in Title

We have found an error in the title of this article, which was recently published in Int. J. Mol. Sci [1]. The correct title should be: Identification of a Functional Oligosaccharide in Maple Syrup as a Potential Alternative Saccharide for Diabetes Mellitus Patients.

## 4. Change in Main Body Paragraphs

In the Abstract:

On page 1, lines 4–6 in the Abstract, the sentence “In the search for such sweeteners, we analyzed the carbohydrate content of maple syrup and identified a novel oligosaccharide composed of fructose and glucose, linked at the C-4 of glucose and the C-6 of fructose” should be replaced by “In the search for such sweeteners, we analyzed the carbohydrate content of maple syrup and identified a functional oligosaccharide. This oligosaccharide has been reported as blastose, which was a disaccharide composed of glucose and fructose with the C-6 of glucose and the C-2 of fructose.”

On page 1, lines 10–12 in the Abstract, the sentence “These findings suggest that this novel oligosaccharide might represent a useful alternative sweetener for inclusion in the diet of patients with DM and may also have therapeutic benefits” should be replaced by “These findings suggest that this functional oligosaccharide might represent a useful alternative sweetener for inclusion in the diet of patients with DM and may also have therapeutic benefits.”

In the Introduction section:

On page 2, lines 11–15 of the original publication [1], the sentences “We successfully identified a novel oligosaccharide in maple syrup that strongly interacts with invertase. Furthermore, we examined what effect this novel oligosaccharide has on the other glycosidases. Therefore, we hypothesize that this novel oligosaccharide has inhibitory activity against glycosidases which, in turn, has beneficial effects on hyperglycemia in DM animal models” should be replaced by “We successfully identified a functional oligosaccharide in maple syrup that strongly interacts with invertase. Furthermore, we examined what effect this functional oligosaccharide has on the other glycosidases. In light of this, we hypothesize that this functional oligosaccharide has inhibitory activity against glycosidases, which, in turn, has beneficial effects on hyperglycemia in DM animal models.”

In the Results section:

*2.2. Purification and Structural Analysis of the Novel Oligosaccharides in Maple Sap*

There are some mistakes in this section of our original publication [1], as follows:

On page 3, the section title “Purification and Structural Analysis of the Novel Oligosaccharides in Maple Sap” should be replaced by “Purification and Structural Analysis of the Unidentified Oligosaccharide in Maple Sap.”

On page 5, the end of paragraph 2 in this section, the authors wish to add the following sentences: “The other fractions obtained by purification were analyzed in the same way; as a result, at least one other unidentified disaccharide was clearly detected. We called these disaccharides ‘maplebioses’ and named the target disaccharide ‘maplebiose1,’ which has an interaction with invertase.”

On page 5, lines 6–9 of this section, the sentences “Subsequently, this oligosaccharide was subjected to NMR analysis to determine its structure more precisely. The proton and carbon signals collected are shown in Table 1. From the ^1^H-^13^C HMBC data, structural formula of this oligosaccharide was predicted as shown in Figure 3. We called this novel oligosaccharide ‘maplebiose1′, and its function was then studied” should be replaced by “Subsequently, maplebiose1 was subjected to NMR analysis to more precisely determine its structure. The proton and carbon signals collected are shown in Table 1. However, from ^1^H–^13^C HMBC as ^13^C and ^1^H signals, no correlation of the linkage between glucose and fructose was obtained. On the other hand, Homann et al. showed NMR spectra data of blastose, which is a disaccharide composed of glucose and fructose [2]. All δ_C_ values of maplebiose1 are close to those of blastose, although uniformly shifted by 0.2 ppm. From these results, maplebiose1 was identified as blastose. The structural formula of maplebiose1 was predicted, as shown in Figure 3. However, blastose has not been enough studied on its function, especially enzyme inhibition. Therefore, we advanced functional studies noticed interaction with invertase for its new applicability.”

In the Discussion section:

On page 7, in the first paragraph of this section, due to incorrect information, replace:

“There is a requirement to restrict the use of sweeteners, such as sucrose and honey, in patients with DM, but such restriction reduces their quality of life. Therefore, the identification of useful alternative sweeteners is likely to be of great interest. In the present study, we have carefully analyzed the carbohydrate composition of maple syrup and found several unidentified saccharides that might have properties suitable for this purpose. In particular, we have identified a novel oligosaccharide in maple syrup that interacts with invertase (Figure 1a–c). Therefore, we aimed to isolate and purify this saccharide to determine its structure and function. Because maple syrup is produced by boiling down maple sap, it is not suitable for the isolation of its components due to its high viscosity. Therefore, we successfully attempted to purify the target oligosaccharide from maple sap, in which it was present at a relatively high concentration. Structural analysis using NMR spectroscopy showed that the target oligosaccharide is a disaccharide composed of fructose and glucose (Figure 2b) with the C-4 of glucose linked to the C-6 of fructose (Figure 3). To our knowledge, this oligosaccharide is a novel saccharide that has a type of glycosidic bond not found in other disaccharides. We have designated this novel oligosaccharide ‘maplebiose1’.”

with

“There is a requirement to restrict the use of sweeteners, such as sucrose and honey, in patients with DM, but such restriction reduces their quality of life. Therefore, the identification of useful alternative sweeteners is likely to be of great interest. In the present study, we carefully analyzed the carbohydrate composition of maple syrup and found several unidentified disaccharides—i.e., ‘maplebioses’—that might have properties suitable for this purpose. In particular, we identified one of the maplebioses, namely, ‘maplebiose1,’ that interacts with invertase (Figure 1a–c). Therefore, we aimed to isolate and purify maplebiose1 to determine its structure and function. Because maple syrup is produced by boiling down maple sap, it is not suitable for the isolation of its components due to its high viscosity. Therefore, we successfully attempted to purify maplebiose1 from maple sap, in which it was present at a relatively high concentration. Structural analysis using NMR spectroscopy showed that maplebiose1 is blastose reported by Homann et al. [2], which is a disaccharide composed of fructose and glucose (Figure 2b) with the C-6 of glucose linked to the C-2 of fructose (Figure 3). On the other hand, blastose was determined as the transfructosylation product of levansucrase [3,4]. However, to the best of our knowledge, it has not been reported that blastose has an inhibitory effect on glycosidases, such as invertase.”

On page 8, in the last paragraph of this section, the sentence “In conclusion, we have isolated maplebiose1, a novel saccharide, from maple syrup and found that it is an invertase inhibitor in the present study” should be replaced by “In conclusion, in the present study, we isolated maplebiose1, one of the unidentified oligosaccharide ‘maplebioses,’ in maple syrup and found that maplebiose1 was blastose reported already. Furthermore, we found that this oligosaccharide has a novel function which is a glycosidase inhibition, and its function restricts glucose absorption in the small intestine.”

In the Material and Methods section:

*4.10. Invertase Inhibition Assay*

On page 10, lines 1–4 of this section, the sentence “The enzymatic reaction was inhibited by adding the novel oligosaccharide, named ‘maplebiose1′, concentrations of 1, 10, or 100 µg/50 µL in 100 mmol/L acetate buffer (pH 4.5) to 100 µg sucrose, and initiated by the addition of 50 µL of 0.2 U/mL invertase solution in water” should be replaced by “The enzymatic reaction was inhibited by adding the functional oligosaccharide, called ‘maplebiose1,’ at concentrations of 1, 10, or 100 µg/50 µL in 100 mmol/L acetate buffer (pH 4.5) to 100 µg sucrose, and initiated by the addition of 50 µL of 0.2 U/mL invertase solution in water.”

*4.13. Oral Sucrose Tolerance Testing*

On page 11, lines 1–3 of this section, the sentence “Sucrose (1.5 g/kg), with or without oligosaccharide (1.62 mg/kg), was administered orally to rats fasted for 14 h, and blood samples were taken from tail veins at subsequent time points for the measurement of plasma glucose (PG) and insulin concentrations” should be replaced by “Sucrose (1.5 g/kg), with or without maplebiose1 (1.62 mg/kg), was administered orally to rats fasted for 14 h, and blood samples were taken from their tail veins at subsequent time points for the measurement of plasma glucose (PG) and insulin concentrations.”

The authors would like to apologize for any inconvenience caused to the readers by these changes.
